# Discovery of Selective Butyrylcholinesterase (BChE) Inhibitors through a Combination of Computational Studies and Biological Evaluations

**DOI:** 10.3390/molecules24234217

**Published:** 2019-11-20

**Authors:** You Zhou, Xin Lu, Hongyu Yang, Yao Chen, Feng Wang, Jifu Li, Zhiran Tang, Xifei Cheng, Yingbin Yang, Li Xu, Qingyou Xia

**Affiliations:** 1 State Key Laboratory of Silkworm Genome Biology, College of Biotechnology, Southwest University, Chongqing 400715, China; 2 School of Pharmacy, China Pharmaceutical University, Nanjing 210009, China; 3 School of Pharmacy, Nanjing University of Chinese Medicine, Nanjing 210023, China; 4 School of Life Science, Southwest University, Chongqing 400715, China

**Keywords:** Alzheimer’s disease, selective butyrylcholiesterase inhibitors, virtual screening, pharmacophore model, acetylcholinesterase, in vitro enzyme assays

## Abstract

As there are increased levels and activity of butyrylcholiesterase (BChE) in the late stage of Alzheimer’s disease (AD), development of selective BChE inhibitors is of vital importance. In this study, a workflow combining computational technologies and biological assays were implemented to identify selective BChE inhibitors with new chemical scaffolds. In particular, a pharmacophore model served as a 3D search query to screen three compound collections containing 3.0 million compounds. Molecular docking and cluster analysis were performed to increase the efficiency and accuracy of virtual screening. Finally, 15 compounds were retained for biological investigation. Results revealed that compounds **8** and **18** could potently and highly selectively inhibit BChE activities (IC_50_ values < 10 μM on human BChE, selectivity index BChE > 30). These active compounds with novel scaffolds provided us with a good starting point to further design potent and selective BChE inhibitors, which may be beneficial for the treatment of AD.

## 1. Introduction

Alzheimer’s disease (AD), an age-related and progressive neurodegenerative disorder featuring memory loss and cognitive impairments, is the most common type of dementia among older adults [1]. It affects more than 30 million patients worldwide at present, and the prevalence of AD continues to increase due to population aging [2,3]. Additionally, the current cost of dementia is about a trillion US dollars a year, and this figure will rise to 2 trillion US dollars by 2030 [4]. Thus, AD has become one of the greatest public health issues worldwide and it severely impacts patients and their families [5].

So far, the pathogenesis of AD still remains unclear, while multiple hypotheses, such as the amyloid-β oligomer hypothesis, the cholinergic hypothesis, and the tau hypothesis, have been offered to explain the mechanism of AD development and set the stage for new therapeutic approaches against AD [6,7,8]. Over the past decade, several anti-AD drugs (tacrine, donepezil, and rivastigmine et al.) targeting the cholinergic dysfunction have been clinically employed for the treatment of AD based on the cholinergic hypothesis, according to which the decline of hippocampal and cortical levels of acetylcholine (ACh) contributes substantially to cognitive decline [9,10,11,12]. In addition, several lines of evidence also indicated that both cholinesterases (ChEs), named as acetylcholinesterase (AChE) and butyrylcholinesterase (BChE), play an important role in amyloid-β (Aβ) aggregation during the senile plaque formation and using the inhibitors can decrease senile plaques [13]. Over the past decade, AChE inhibitors have been investigated extensively as anti-AD agents. However, the therapeutic effect of AChE inhibitors is limited, while side effects like nausea and vomiting induced by undesirable inhibition of peripheral ChEs also hinder the long-term application in clinical trials [14,15]. Recently, increasing evidence have indicated that BChE plays a pivotal role in regulating brain ACh level in the late stage of AD. In progressed AD, the AChE level in the brain decreases to 55%–67% of normal values while BChE increases to 165% of normal levels [16,17]. Moreover, AChE knockout mice models indicated that BChE can potentially substitute for AChE, maintaining normal cholinergic pathways in AChE nullizygous animals [18]. In addition, BChE inhibition is not accompanied by the peripheral adverse effects [19]. Therefore, development of highly potent and selective BChE inhibitors that are able to restore Ach levels in the brain, with much reduced peripheral side effects, represents a significant advancement [20].

**Figure 1:**
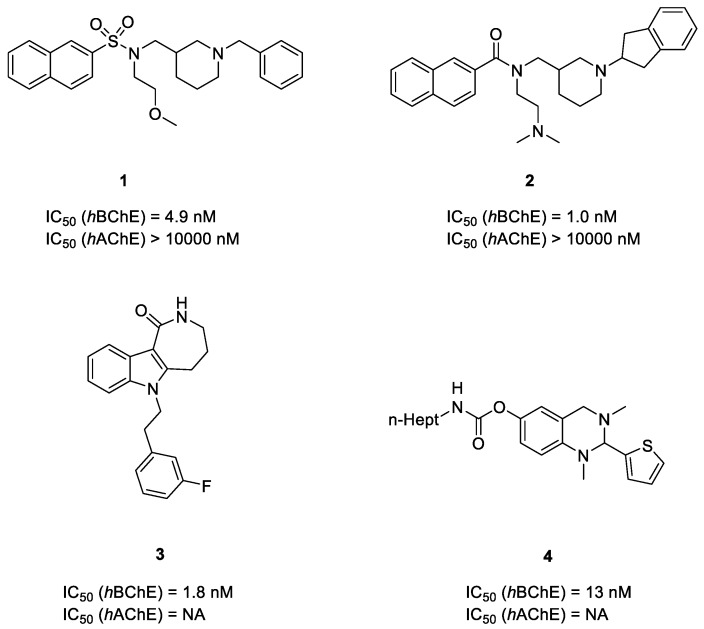
Recently reported selective human butyrylcholinesterase (*h*BChE) inhibitors.

Up to now, only a limited number of highly selective and potent BChE inhibitors have been reported, as the two isoforms human AChE (*h*AChE) and human BChE (*h*BChE) are highly homologous proteins [21] (Figure 1). Stanislav Gobec et al. reported a series of sulfonamide BChE inhibitors in 2016. Among them, compound **1** showed highly selective and nanomolar inhibitory activity against *h*BChE [22]. Further modification of compound **1** led to the generation of naphthamide compound **2** with slightly improved potency [23]. Additionally, 6-substituted 3,4,5,6-tetrahydroazepino[4,3-*b*]indol-1(*2H*)-one (THAI) compound **3** and 2-thiophenyl compound **4** were also highly potent and selective *h*BChE inhibitors [24,25]. Since BChE-targeting inhibition represents a promising therapeutic approach against AD, the discovery of potent and selective BChE inhibitors is urgent. Over the past decade, multiple X-ray crystallographic structures of complexes between *h*BChE proteins and *h*BChE inhibitors with various scaffolds have been determined, which sets the stage for virtual screening [26,27,28]. There are already several reports of using a structure-based virtual screening protocol and successfully obtained potent and highly selective *h*BChE inhibitors [16,29,30]. After identifying drug hits with novel scaffolds and desired properties by virtual screening, further studies like structure–activity relationship (SAR) studies based on enzyme or cell bioassay and crystallographic study could provide more useful information for structural modification and improvement [31,32,33].

**Figure 2:**
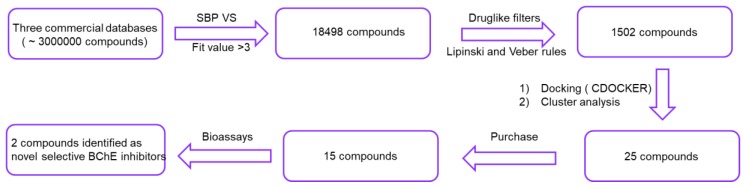
An overview of the structure-based pharmacophore virtual screening (SBP VS) protocol applied to identify selective butyrylcholinesterase (BChE) inhibitors.

Recently, Sun et al. reported a valid structure-based pharmacophore model which led to the discovery of three compounds displaying IC_50_ values < 2 μM on *h*BChE [34]. Considering the effectiveness of the pharmacophore model, further structure-based pharmacophore virtual screening (SBP VS) of three commercial compound collections (ChemBridge, ChEMBL, Vitas-M) was reported in this paper. The employed screening workflow was depicted in Figure 2. With the integration of docking and diversity analysis, 25 compounds were selected and 15 of them were purchased for bioassays. Finally, two compounds with different scaffolds were identified as selective *h*BChE inhibitors. The new emerging molecules with different scaffolds not only enriched the structural types of selective BChE inhibitors, but also may provide valuable starting structures for medicinal chemists to develop anti-AD drugs.

## 2. Results and Discussion

### 2.1. Virtual Screening

**Figure 3:**
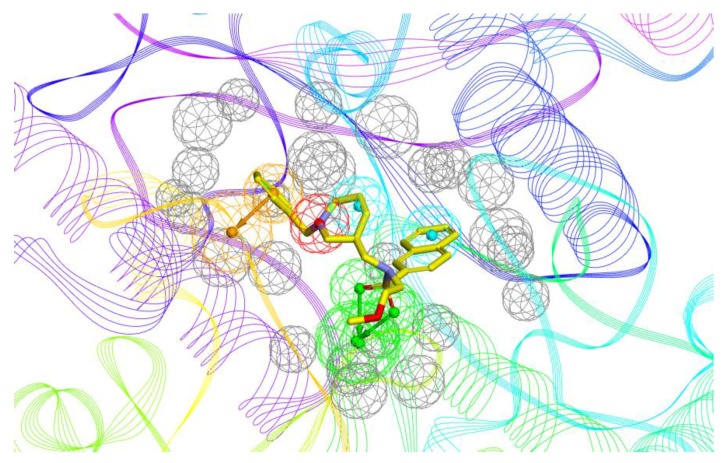
The pharmacophore model bound with **1**-BChE complex. The Compound is represented in yellow stick mode. In the pharmacophore model, the hydrogen bond acceptor, hydrophobic portion, aromatic ring, cation, and excluded volumes are colored green, cyan, orange, red, and grey, respectively.

**Figure 4:**
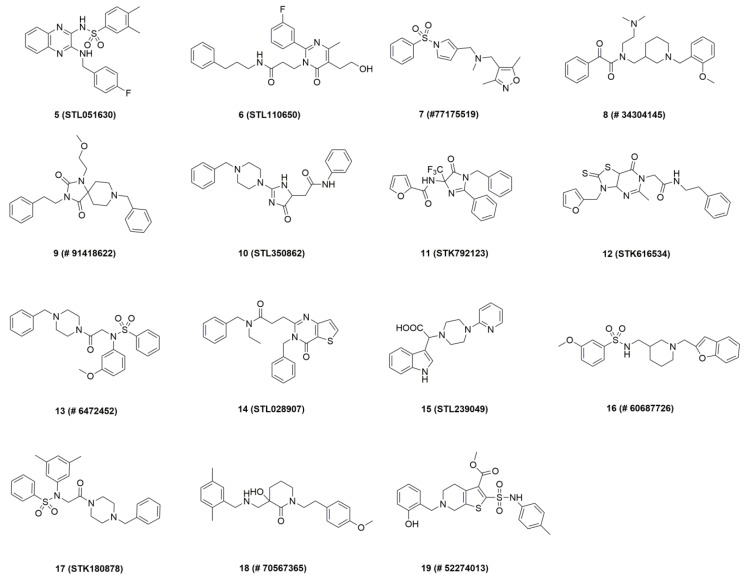
Chemical structure of the compounds purchased for in vitro tests.

The construction and validation of the 3D pharmacophore model was reported in the previous paper [34]. Generally, the six-feature model was generated on the basis of **1**-BChE complex (PDB ID: 5DYW) [22]. Specifically, the model consists of two hydrogen bond acceptor points, two hydrophobic points, one aromatic ring point, and one positive ionizable point (Figure 3). In this paper, it was used for virtual screening of three commercial compound collections: Vitas-M (1315684), ChEMBL (918887), ChemBridge (777451). We retained 18,498 compounds whose pharmacophore fit value above 3, after the first filtration step. Next, 1502 compounds with favorable physicochemical properties were identified by using drug-like descriptors of Lipinski and Veber rules [35]. In order to reduce the amount of compounds to handle prior to biological experiments, two complementary methods including docking and structural clustering were utilized. On one hand, the CDOCKER module of Discovery Studio 3.0 (DS) was applied to predict the binding modes of all selected virtual hits with BChE active site (PDB ID: 5DYW) to check their interactions with BChE binding site residues [36]. The docked compounds were ranked by the corresponding values of –CDOCKER energy. Careful visual inspection was performed, and the following interactions were considered significant: π–π interaction between the molecule and Trp231, Trp82, and Phe329, H-bonding interaction of the molecule with His438, and occupancy of the molecule in the acyl pocket. A set of 100 compounds were saved. On the other hand, the above-mentioned 1502 compounds were subjected to structural clustering. Ten clusters were produced based on the FCFP_6 fingerprints using the Cluster Ligand module of DS and twenty compounds with diverse scaffolds in each cluster were chosen. In consideration of the binding mode and structural diversity, 25 candidates were selected and 15 commercially available candidates (Figure 4) were purchased from Topscience (www.tsbiochem.com) for biological investigation.

### 2.2. ChEs Inhibitory Activities of Hit Compounds

Initial screening of the 15 potential inhibitors obtained from in silico studies was performed with human ChE*s* using a modified Ellman’s assay, and tacrine was used as the reference control (Table 1). The result indicated that compounds **8** and **19** exhibited over 50.0% inhibitory effects on both AChE and BChE at the concentration of 10 μM. Interestingly, compound **18** exhibited selective BChE inhibitory effect (BChE = 58.4% at 10 μM, AChE = 11.1% at 10 μM). Next, the dose-dependent inhibitory activities of compounds 8, 18, and 19 against BChE and AChE were tested at doses ranging from 10^−4^ to 10^−9^ M, and their IC_50_ values were calculated (Figure S1). The result demonstrated that three compounds showed great anti-BChE activities (BChE IC_50_ < 10 μM). Additionally, compounds **8** and **18** showed much better BChE selective index (SI BChE, AChE IC_50_/BChE IC_50_ > 30) than compound **19** (SI BChE = 6). To the best of our knowledge, compounds **8** and **18** were structurally different from the previously reported selective BChE inhibitors, and were used in the follow-up studies.

### 2.3. Kinetic Studies

**Figure 5:**
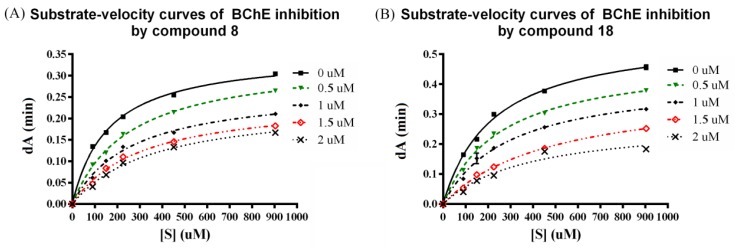
Representative plot of BChE activity and the effect of substrate concentration (90–904 μM) in the absence of inhibitor and in the presence of **8** and **18** (0.5–2 μM). (**A**) Substrate-velocity curves of BChE inhibition by compound **8**; (**B**) Substrate-velocity curves of BChE inhibition by compound **18**.

As compounds **8** and **18** showed selective BChE inhibitory activity, they were selected to perform enzymatic kinetic studies with BChE in order to gain information about the mode of inhibition and binding. As shown in Figure 5, the patterns clearly indicate both compounds are mixed-type inhibitors: The presence of compounds **8** and **18** reduce the maximum velocity ***V*_m_**, and increase the *K*_m_ value. This means that compounds **8** and **18** can bind to the free enzyme, and to the Michaelis complex of the enzyme and substrate. The inhibition constant *K*i values of **8** and **18** are shown in Table 2.

### 2.4. Docking Simulation of Hit Compounds

**Figure 6:**
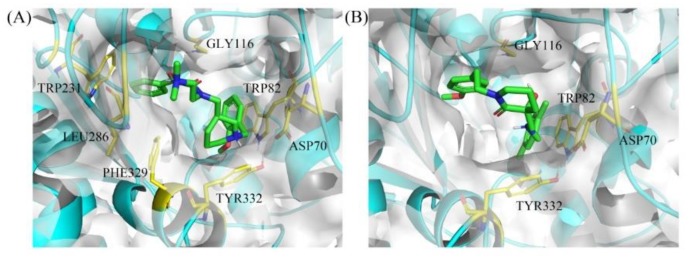
Binding mode predictions for compound **8** (**A**) and **18** (**B**) with BChE domain (PDB ID: 5DYW). Compounds were shown in green stick mode; key residues were shown in yellow stick mode; the solvent accessible surface (SAS) of proteins contoured by white.

To verify the binding mode of hit compounds **8** and **18** to BChE, we carried out a docking simulation using CDOCKER module in DS 3.0 and the docking results are shown in Figure 6.

For **8** (Figure 6A), the phenyl group occupies the acyl binding pocket (mainly formed by Trp231, Leu286, and Val288) and interacts with Trp231 via π–π T-shaped interaction. In addition, the positively charged nitrogen of piperidine moiety forms a salt bridge with Asp70 in the peripheral anionic site (PAS) and π–cation interaction with Tyr332, respectively. Basic or permanently charged nitrogens are proposed as important for cation–π interactions with active site residues of ChE and blood-brain barrier (BBB) permeability, and common amidst ChE inhibitors [37,38,39,40]. The 2-methoxybenzyl group fits into the choline binding pocket (mainly formed by Tyr332 and Trp82), which further enhances the binding affinity. Furthermore, the -(CH_2_)_2_-N(Me)_2_ side chain points out of the gorge and stabilizes the U-shaped conformation.

For **18** (Figure 6B), although the structure is obviously different from **8**, the binding pattern of **18** with BChE, including the orientation of the binding pose and the key residue for the intermolecular interaction, is similar to that of **8** with BChE. However, the inhibitory activity of **18** towards BChE is lower than **8**, this could be explained by the following reasons: (1) The protonation ability of the secondary amine is lower than the tertiary amine; and (2) the 4-substituted benzyl ring of **18** is not completely inserted into acyl binding site, therefore, it is unable to interact with Trp231, which plays an important role in BChE–ligand interaction [41].

### 2.5. Molecular Dynamics

To further understand the binding modes of **8** and **18** with BChE, we explored the interactions between BChE and compounds by carrying out 100 ns molecular dynamics (MD) simulations for the complexes of **8**-BChE and **18**-BChE. Stable MD simulations trajectories were utilized for data extraction and binding free energy calculation. Time dependencies of root-mean-squared deviation (RMSD) values for the backbone atoms of proteins and compounds during the MD simulation were provided in Figure S2. The total free binding energies were calculated using the molecular mechanics/Poisson–Boltzmann surface area (MM-PBSA) method. As shown in Table 3, the total binding free energy was −42.90 ± 9.39 and −48.23 ± 6.08 kcal/mol for the complexes of BChE-**8** and BChE-**18**, respectively. The result indicated that the binding of the two compounds with BChE was energetically stable. It is noteworthy that the electrostatic energy (EEL) played a greater role in the binding of the two compounds with BChE than the hydrophobic contacts (van der Waals energy (VDWAALS)).

**Figure 7:**
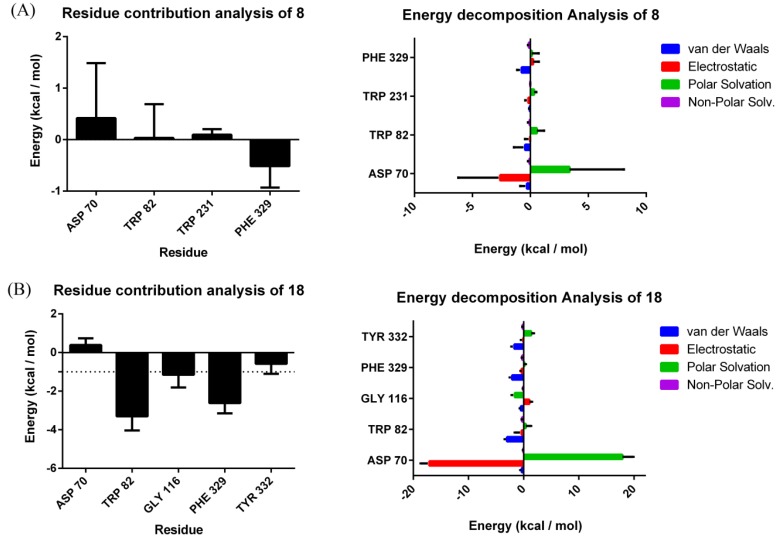
Residue contributions and energy decomposition of potential hot residues of **8** (**A**) and **18** (**B**); for all energies, unit is kcal/mol.

The contribution of potential hot residues for the binding of **8** and **18** was evaluated with the MM-PBSA method (Figure 7). Meanwhile, energy decomposition of potential hot residues was performed to evaluate which interaction was the dominating factor for the binding free energy (Figure 7). Usually, a residue is supposed to be vital for interaction of proteins with ligands if the interaction energy with ligand is lower than −1 kcal/mol [42]. For **8**, none of the determined residues (Asp70, Trp82, Trp231, and Phe329) are the key residue for the binding of **8** with BChE. This result could be explained by the significant conformational change of **8** during MD process, which might be related with the presence of retained water molecules (Figure S2). For **18**, Trp82 (−3.30 ± 0.35 kcal/mol), Gly116 (−1.15 ± 0.66 kcal/mol) and Phe329 (−2.61 ± 0.53 kcal/mol) were considered as important residues for binding of 18 with BChE.

**Figure 8:**
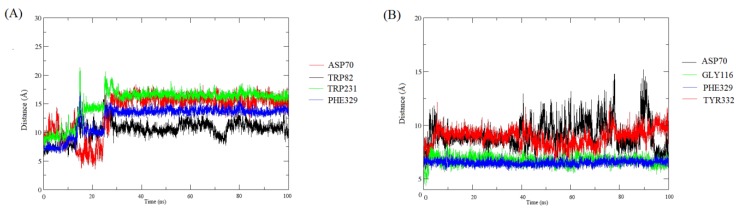
The evolution of interatomic distances between chemical functions of the ligands and key amino acid residues in the binding pocket during molecular dynamics (MD). (**A**) The evolution of interatomic distances between chemical functions of **8** and key amino acid residues during MD. Asp70 represented the distance between N atom of the piperidine ring and Asp70; Trp82 represented the distance between the methoxy-substituted benzene ring and Trp82; Trp231 represented the distance between the unsubstituted benzene ring and Trp231; (**B**) The evolution of interatomic distances between chemical functions of **18** and key amino acid residues during MD. Asp70 represented the distance between N atom of the secondary amine group and Asp70; Gly116 represented the distance between the methoxy-substituted benzene ring and Gly116; Phe329 represented the distance between the methoxy-substituted benzene ring and Phe329; Tyr332 represented the distance between N atom of the secondary amine group and Tyr332.

The evolution of interatomic distances between functional groups of ligands and the above-mentioned key amino acid residues during MD was also performed to obtain more detailed information. As shown in Figure 8A, the distances between the functional groups of **8** and four monitored amino acid residues in the binding pocket: the piperidine ring-Asp70, the benzene ring-Trp82, the benzene ring-His231, and the benzene ring–Phe329, seemed to be changing greatly during the MD simulation, showing that these interactions are relatively unstable. In contrast, the distances between the benzene ring of **18** and important amino acid residues (Phe329 and Gly116) basically maintained the same during the MD simulation. However, the distances between compound **18** and two amino acid residues, Asp70 and Trp332, change greatly. This might explain why Asp70 and Trp332 played a minor role in the binding of **18** with BChE. All these MD results provide detailed information on the interactions between the hits and BChE.

### 2.6. Cell Viability Assay

**Figure 9:**
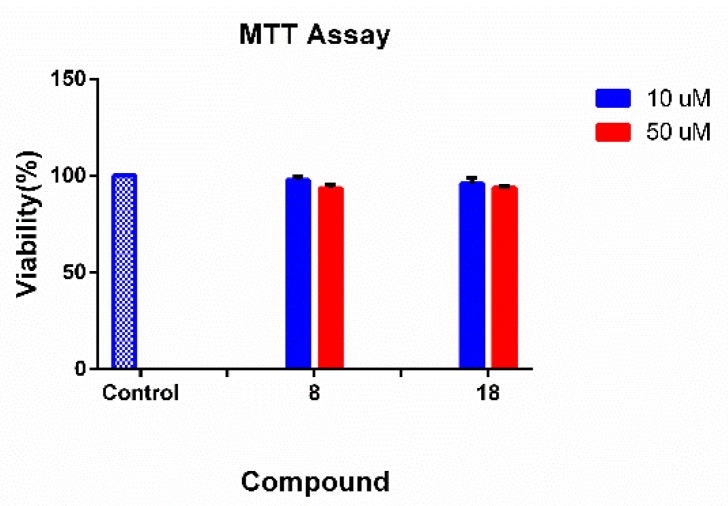
The cytotoxicity of hit compounds on SH-SY5Y cells.

The 3-(4,5-dimethylthiazol-2-yl)-2, 5-diphenyltetrazolium (MTT) assay was used to evaluate potential cytotoxic effects of hit compounds **8** and **18** on neuroblastoma cell line SH-SY5Y [43]. As indicated in Figure 9, none of compounds was observed to affect cell viability at concentrations of 10 μM and 50 μM. The result indicated that 8 and 18 have preliminary safety on neuroblastoma cell line SH-SY5Y.

### 2.7. Absorption, Distribution, Metabolism, Excretion, and Toxicity (ADMET) in Silico Prediction

As shown in Table 4, compounds **8** and **18** appeared to have very poor solubility in aqueous media which have already been observed during bioassays. Both compounds were predicted to possess good absorption properties, which means both compounds could successfully enter the blood circulation from the site of administration. Additionally, compounds **8** and **18** were predicted to have high and medium blood–brain barrier (BBB) penetration, respectively, which is vital for AD treatment. The polar surface area (PSA-2D) of both compounds was less than 80. Notably, **8** may bind to CYP2D6, which would affect the efficacy of **8** and result in potential side effects. All compounds may be highly bound to plasma proteins. In this prediction, both compounds were negative in hepatotoxicity. In brief, further biological experiments are required to provide additional data.

## 3. Materials and Methods

### 3.1. Virtual Screening

The validated pharmacophore model, consisting of two hydrogen bond acceptor points, two hydrophobic points, one aromatic ring point, and one positive ionizable point, was used to screen three commercial databases using Accelrys Discovery Studio 3.0 (DS, Accelrys, Inc. San Diego, CA, USA). Virtual screening was performed based on the query fit. The drug-likeness of compounds was assessed using the DS 3.0 Filters ligands using Lipinski and Veber Rule protocol. Docking simulation was performed with the CDOCKER module implemented in DS, using the same protein applied for pharmacophore generation. A site sphere (in 10 Å radius) around the native compound was defined as the binding site. Other parameters were set as the defaults. The Cluster Ligand Module was used to cluster compounds. The number of cluster was set as 10 and the predefined set was FCFP_6. Other parameters were maintained as the defaults. Finally, 15 hits were purchased from Topscience (www.tsbiochem.com), with purity >95% (liquid chromatography-mass spectrometry, LC-MS).

### 3.2. Biological Evaluation

Modified Ellman’s assay was performed to measure the inhibitory effects of purchased compounds on ChEs using a Thermo Fisher Scientific spectrophotometer [44]. AChE (from human erythrocytes, C0663), BChE (from human serum, B4186), 5,5-dithiobis-(2-nitrobenzoic acid) (DTNB, D218200), acetylthiocholine iodide (ATC, A5751), and butyrylthiocholine iodide (BTC, B3253) were purchased from Sigma-Aldrich (St. Louis, MO, USA).

Each compound was dissolved in DMSO and prepared a dilution series of six different concentrations (10^−4^ to 10^−9^ M) that the DMSO concentrations lower than 1%. For measurement, a cuvette containing 30 μL of phosphate buffer, 10 μL of AChE (2.5 units/mL) or BChE (2.5 units/mL), and 10 μL of the test compound solution was allowed to stand for 5 min before 10 μL of DTNB was added. After the addition of 20 μL of ATC or BTC, the reaction was initiated and the solution was mixed immediately. Two minutes after substrate addition, the absorption was measured at 412 nm by Thermo Fisher Scientific spectrophotometer (multiskan FC, USA). 10 μL of phosphate-buffered solution replaced the enzyme solution were used to determine the blank value. All the measurements were done in triplicate. The inhibition curve was drawn by plotting the percentage enzyme activity (100% for the reference) versus the logarithm of the test compound concentration. The IC_50_ values were calculated by GraphPad Prism 6 (GraphPad Software, San Diego, CA, USA), and the data were shown as mean ± standard error of mean (SEM).

### 3.3. Kinetic Study

Kinetic studies were performed in the same manner as the determination of ChE inhibition as previously described [45]. The substrate (BTC) was used at various concentrations (90, 150, 226, 452, and 904 μM) for each test compound concentration and the enzymatic reaction was extended to four minutes before determining the absorption. The *V*_max_ and *K*_m_ values of the Michaelis–Menten kinetics were calculated by nonlinear regression from substrate–velocity curves using Graphpad Prism 6 (GraphPad Software, San Diego, CA, USA) (Table S1). Inhibition constants were evaluated from the effect of substrate concentration (S) on the degree of inhibition according to equation:

*V*_0_ = *V*_max_[S]/(*K*_m_(1 + [I]/*K*ic) + [S] (1 + [I]/*K*iu)) (1)

where S is the substrate BTC, I is the inhibitor, *K*ic is the enzyme–inhibitor inhibition constant of a complex formed at the catalytic site, *K*iu is the Michaelis complex–inhibitor inhibition constant of a complex formed at the peripheral site, *K*_m_ is the Michaelis complex, and *V*_m_ is maximal velocity.

### 3.4. Binding Mode Prediction

The molecular docking procedure to study the binding mode of compounds **8** and **18** against BChE was performed according to our previously reported protocol [36]. The co-crystal structure of *N*-((1-benzylpiperidin-3-yl)methyl)-*N*-(2-methoxyethyl)naphthalene-2-sulfonamide–BChE (PDB ID: 5DYW) was used here.

### 3.5. Molecular Dynamics

MD simulations were performed using the Particle Mesh Ewald Molecular Dynamics (PMEMD) module in AMBER 16 accelerated by GPU system consisting of the NVIDIA CUDA processor [46]. Five structural water molecules (PDB ID: 5DYW, Chain B: HOH806, HOH765, HOH749, HOH761, and HOH715) were conserved during the preparation of the protein model for molecular docking (Figure S3). The other steps were the same as usual. Briefly, the 10 top ranked binding poses were retained after molecular docking. The structure, from which its ligand had interactions with known crucial amino acids like Trp82, Trp231, and Phe329 et al., was selected for the next MD simulation. The proteins were assigned with the AMBER ff99SB force field, while the ligands were treated with the ANTECHAMBER module and the general AMBER force field [47,48]. All hydrogen atoms of proteins and ligands were added using the reduce module. The systems were solvated in a TIP3P water box in a 9 Å hexahedron. Sodium ions were added to neutralize the systems. Each system was subjected to 1000 steps of steepest-descent energy minimization followed by 1000 steps of conjugate gradient minimization with the purpose of remove possible steric stresses. Both systems were gradually heated from 0 to 300 K using a Langevin thermostat during the initial 60 ps and weak restraints 10 kcal/mol on the protein backbone atoms over 1 ns. Finally, a dynamics simulation of 20 ns NPT ensemble was set at 1 atm and 300 K. After MD simulation, the trajectory was stored every 1 ps for CPPTRAJ analysis [49]. Binding free energies and energy decomposition was determined by using the MM-PBSA method in the AMBER 16 [50]. The distance between chemical functions and residues were monitored within CPPTRAJ module. The chemical group was defined as centroid of multiple atoms. Specifically, the phenyl group was defined using the center of its six carbon atoms. The results were shown in XMGRACE.

### 3.6. Cell Viability Assay

The human neuroblastoma SH-SY5Y cells (3 × 10^3^, DMEM medium supplemented with 10% fetal bovine serum, volume 0.1 mL) were placed in a 96-well flat-bottomed plate, in a humidified atmosphere of 95% air and 5% CO_2_ at 37 °C and grown to 80% confluence. Before cell treatment, the complete medium was replaced with reduced-serum medium (BI, 01-052-1ACS) [43]. Then, cells were treated with various concentrations of compounds for 24 h. MTT reagent (D&B, Q108115) (0.5 mg/mL) was added to the wells and the plates were incubated for 4 h at 37 °C. Supernatants were carefully removed, and 150 μL of dimethylsulfoxide (DMSO) were added into each well. The absorbance was measured at 570 nm using the spectrophotometer (Thermo, multiskan FC, USA). Cells were treated in quadruplicate. The values are the mean ± SD of three independent experiments.

### 3.7. ADMET in Silico Prediction

The ADMET properties (absorption, distribution, metabolism, excretion, and toxicity) and physicochemical properties of compounds **8** and **18** were calculated within ADMET software and Calculate Molecular Properties in Discovery Studio 3.0.

## 4. Conclusions

Considering the effectiveness of the pharmacophore model built by Sun et al. in the previous study, further pharmacophore-based virtual screening of three commercial compound collections containing 3.0 million compounds was reported in this paper to identify new BChE inhibitors [34]. Molecules passed pharmacophore filters and drug-like filters were further studied by using two complementary methods including docking and cluster to select potential hit compounds for biological evaluation. Satisfyingly, in vitro enzyme inhibition tests confirmed that compounds **8** and **18** could potently and highly selectively inhibit BChE activities (BChE IC_50_ < 10 μM and SI BChE > 30). In addition, the result of cell viability assay indicated that compounds **8** and **18** have preliminary safety on neuroblastoma cell line SH-SY5Y at concentrations of 10 μM and 50 μM. Furthermore, MD simulation, docking studies, and kinetic studies were performed to analyze the detailed binding modes of representative compounds **8** and **18** with BChE. The results indicated a mixed-type inhibition of the two compounds towards BChE. These active compounds with novel scaffolds provided us with a good starting point to further design potent and selective BChE inhibitors, which may benefit the treatment of AD.

## Figures and Tables

**Table 1:** The inhibitory activities against cholinesterases (ChEs) of the hits from virtual screening.

Compound		BChE	AChE	
	IR ^a^ (%)	IC_50_^b^ (μM)	IR ^c^ (%)	IC_50_ (μM)
**5**	7.2 ± 0.6	nd. ^d^	−0.31 ± 0.5	nd.
**6**	8.5 ± 0.3	nd.	−1.5 ± 0.5	nd.
**7**	16.3 ± 1.1	nd.	0.6 ± 0.6	nd.
**8**	68.6 ± 0.7	1.1 ± 0.6	58.5 ± 1.2	43.2 ± 17.6
**9**	15.5 ± 1.6	nd.	16.0 ± 1.5	nd.
**10**	9.9 ± 1.0	nd.	7.8 ± 0.7	nd.
**11**	14.8 ± 1.3	nd.	−0.7 ± 0.7	nd.
**12**	−1.8 ± 1.1	nd.	1.1 ± 1.0	nd.
**13**	20.1 ± 1.2	nd.	11.3 ± 1.3	nd.
**14**	3.4 ± 0.4	nd.	10.9 ± 0.8	nd.
**15**	−0.6 ± 0.5	nd.	0.6 ± 1.0	nd.
**16**	26.4 ± 1.1	nd.	38.7 ± 1.7	nd.
**17**	11.8 ± 1.2	nd.	2.9 ± 0.5	nd.
**18**	58.4 ± 0.9	6.3 ± 2.0	11.1 ± 1.5	nd.
**19** **Tacrine**	61.2 ± 1.8 100	2.4 ± 1.0 0.003 ± 0.004	53.2 ± 0.6 95.2 ± 0.3	13.8 ± 6.0 0.01 ± 0.003

All data are shown as mean ± SEM of three experiments. SEM = standard error of mean. ^a^ Inhibition ratio (IR) against AChE at 10 μM. ^b^ IC_50_ values represent the concentration of inhibitor required to decrease enzyme activity by 50%. ^c^ Inhibition ratio (IR) against BChE at 10 μM. ^d^ nd = not determined.

**Table 2:** The inhibition constants for the inhibition of BChE by compounds **8** and **18**.

Compound	*K*ic ^a^	*K*iu ^b^
**8**	0.88 ± 0.07 µM	3.61 ± 0.24 µM
**18**	0.93 ± 0.13 µM	2.31 ± 0.32 µM

All data are shown as mean ± SEM of three experiments. ^a^ *K*ic is the inhibition constant for the competitive part of inhibition. ^b^ *K*iu is the inhibition constant for the uncompetitive part of inhibition.

**Table 3:** Predicted binding free energies (kcal/mol) for bindings of **8** or **18** with BChE by the molecular mechanics/Poisson–Boltzmann surface area (MM-PBSA) method.

Energy Terms (kcal/mol)	8	18
VDWAALS ^a^	−43.3 ± 5.8	−52.2 ± 3.5
EEL ^b^	−51.3 ± 16.8	−142 ± 24.0
EGB ^c^	57.6 ± 15.0	153 ± 21.3
ESURF ^d^	−5.9 ± 1.0	−7.1 ± 0.7
DELTA G gas ^e^	−94.6 ± 19.1	−195 ± 24.6
DELTA G solv ^f^	51.7 ± 14.2	146 ± 20.8
DELTA TOTAL ^g^	−42.9 ± 9.4	−48.2 ± 6.1

^a^ van der Waals energy. ^b^ Electrostatic energy. ^c^ Polar solvation energy. ^d^ Non-polar solvation energy. ^e^ Total gas phase free energy. ^f^ Total solvation free energy. ^g^ Total binding free energy.

**Table 4:** Predicted pharmacokinetic properties of hits.

Comp.	AlogP98 ^a^	PSA-2D ^b^	Solubility Level ^c^	Absorption Level ^d^	BBB Level ^e^	PPB ^f^	CYP2D6 ^g^	Hepatotoxic ^h^
**8**	3.53	53.589	3	0	1	true	true	false
**18**	3.514	63.209	3	0	2	true	false	false

^a^ AlogP98: Lipophilicity descriptor. ^b^ PSA-2D: Polar surface area. ^c^ Solubility Level: (0, good; 1, moderate; 2, poor; 3, very poor). ^d^ Absorption Level: (0, good; 1, moderate; 2, poor; 3, very poor). ^e^ blood–brain barrier (BBB) Level: (0, very high blood–brain barrier penetration; 1, high; 2, medium; 3, low). ^f^ PPB Prediction: PPB refers to plasma protein binding. The classification describing whether a compound is highly bound (>= 90% bound) to plasma proteins using the cutoff Bayesian score of −2.209 (obtained by minimizing the total number of false positives and false negatives). ^g^ CYP2D6 Prediction: The classification describing whether a compound is a cytochrome P450 2D6 (CYP2D6) inhibitor using the cutoff Bayesian score of 0.161 (obtained by minimizing the total number of false positives and false negatives). ^h^ Hepatotoxic Prediction: The classification describing whether a compound is hepatotoxic using the cutoff Bayesian score of −4.154 (obtained by minimizing the total number of false positives and false negatives).

## Supplementary Materials

The following are available online at https://www.mdpi.com/1420-3049/24/23/4217/s1, Figure S1. The primary human ChEs IR (%) screening of hit compounds under 10 μM (**A** and **B**); The dose-dependent inhibition of hits against human BChE (**C**) and human AChE (**D**), respectively. Figure S2: Time dependencies of RMSDs for the heavy atoms (Cα, C, and N) of proteins and ligands. Figure S3. Schematic representation of interaction between ligands (compounds **8** and **18**) and BChE before the MD simulation. Figure S4. The binding modes of ligands (compounds **8** and **18**) with BChE (PDB ID: 5DYW). Protein models were taken from the last frame of the MD simulation. Table S1. The apparent *V*max and *K*m values for compounds **8** and **18** in kinetic studies.
