# Supplementary material for: Discovery of Selective Butyrylcholinesterase (BChE) Inhibitors through a Combination of Computational Studies and Biological Evaluations
Source: Molecules. 2019 Nov 20;24(23):4217. doi: 10.3390/molecules24234217 (PMC6930573; doi:10.3390/molecules24234217)
Supplement: Supplementary file 1 [file molecules-24-04217-s001.pdf]

Discovery of Selective Butyrylcholinesterase (BChE) Inhibitors through a Combination of Computational Studies and Biological Evaluations

You Zhou [1,*], Xin Lu [2], Hongyu Yang [2], Yao Chen [3], Feng Wang [1], Jifu Li [1], Zhiran Tang [4], Xifei Cheng [1], Yingbin Yang [4], Li Xu [1] and Qingyou Xia [1,*]

[1] State Key Laboratory of Silkworm Genome Biology, College of Biotechnology, Southwest University, Chongqing 400715, China; zhouy701005@swu.edu.cn (Y.Z.); wangf1986@swu.edu.cn (F.W.); lijifu0103@163.com (J.L.); chengxifei123456@126.com (X.C.); mulberry@swu.edu.cn (L.X.); xiaqy@swu.edu.cn (Q. X.)

[2] School of Pharmacy, China Pharmaceutical University, Nanjing 210009, China; 1621010068@stu.cpu.edu.cn (X.L.); 1721010040@stu.cpu.edu.cn (H.Y.); sunhaopeng@163.com (H.S.)

[3] School of Pharmacy, Nanjing University of Chinese Medicine, Nanjing 210023, China; clarissa0710@163.com

[4] School of Life Science, Southwest University, Chongqing 400715, China; tangzhiran2007@foxmail.com (Z.T.); yybin@swu.edu.cn (Y.Y.)

* Correspondence: zhouy701005@swu.edu.cn (Y.Z.); xiaqy@swu.edu.cn (Q. X.)

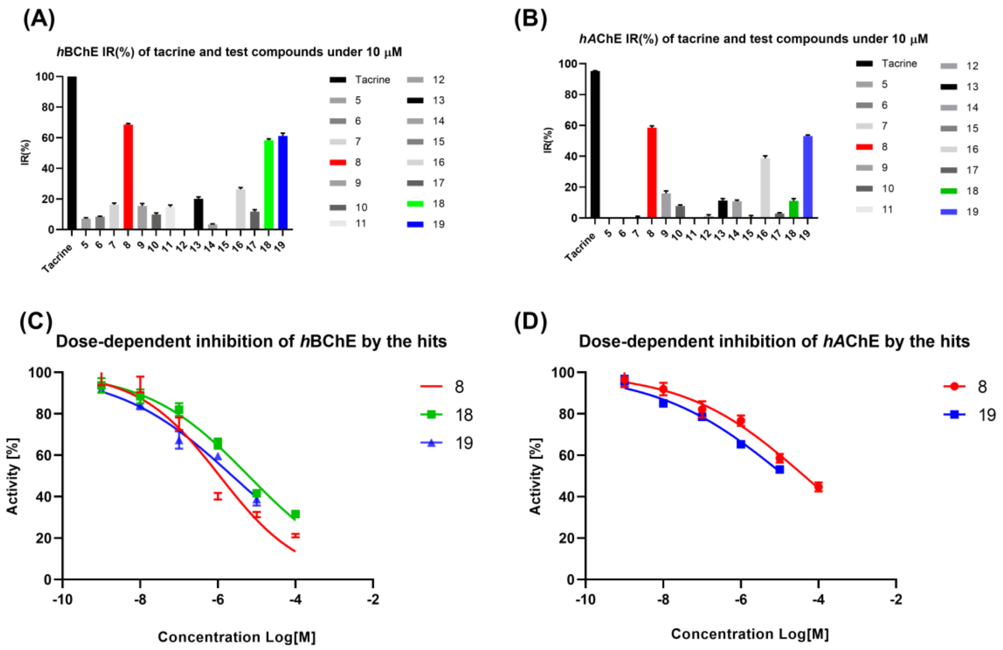

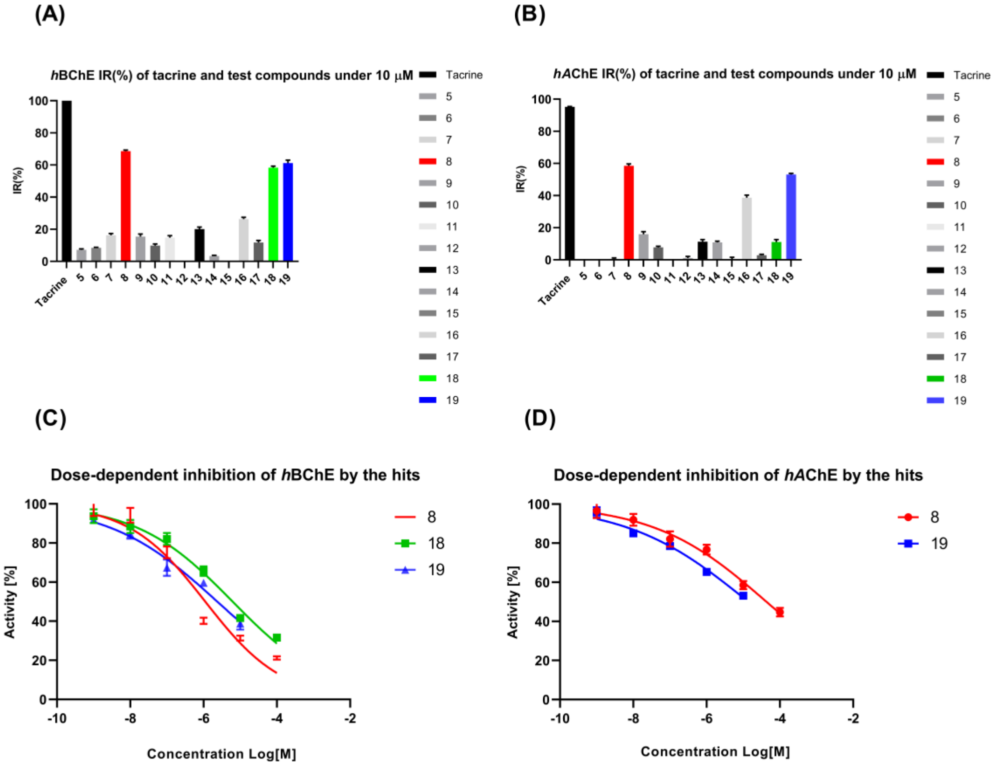

Figure S1. The primary human ChEs IR(%) screening of test compounds under 10 μM (A and B); The dose-dependent inhibition of hits against human BChE (C) and human AChE (D), respectively.

Table S1. The apparent V_{max} and K_m values for compounds **8** and **18** in kinetic studies.

Concentration (μM)	V_{max} (μM min^{-1})	K_m (μM)
	**8 against BChE**	
0	0.35 ± 0.01	160.8 ± 7.9
0.5	0.34 ± 0.01	254.3 ± 10.9
1	0.27 ± 0.01	262.6 ± 19.3
1.5	0.25 ± 0.01	304.4 ± 23.1
2	0.24 ± 0.01	359.8 ± 30.0
	**18 against BChE**	
0	0.57 ± 0.01	224.1 ± 12.6
0.5	0.49 ± 0.01	257.9 ± 14.0
1	0.42 ± 0.02	291.3 ± 33.2
1.5	0.39 ± 0.01	481.5 ± 27.9
2	0.28 ± 0.02	381.3 ± 76.1

Data are shown as the mean ± SD of three experiments.

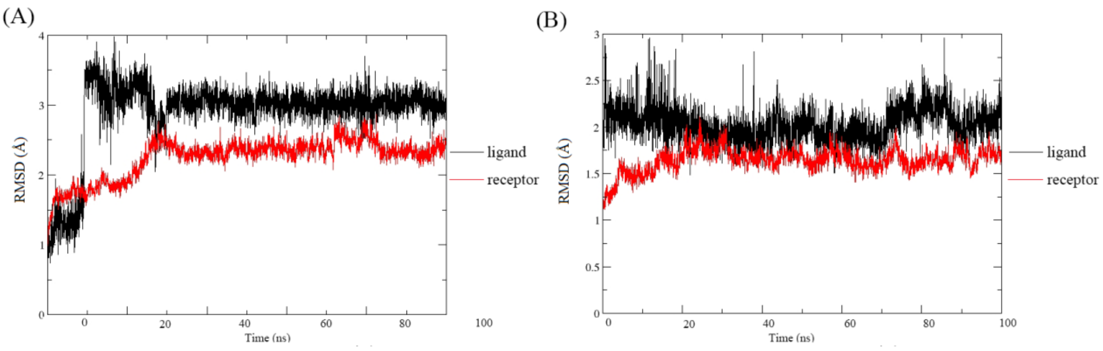

Figure S2. Time dependencies of RMSDs for the heavy atoms (Cα, C and N) of proteins and ligands. (A) **8**-*h*BChE; (B) **18**-*h*BChE.

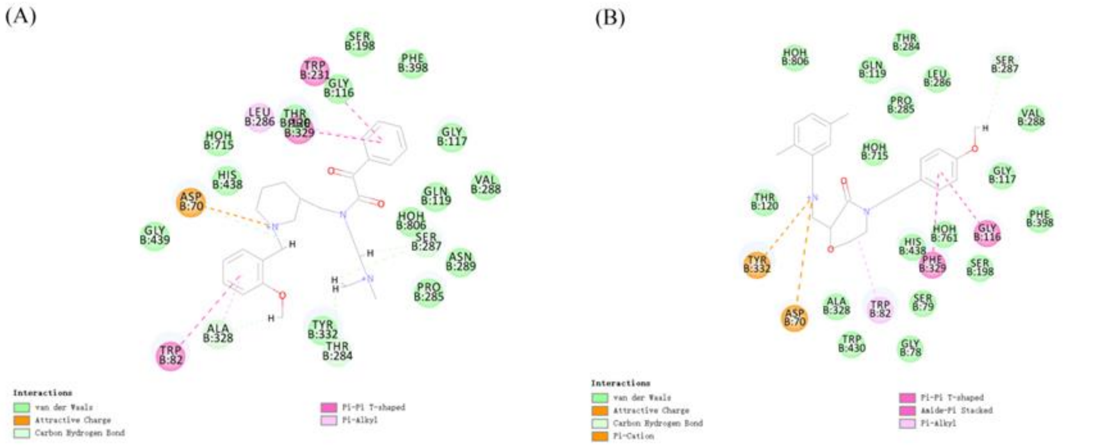

Figure S3. Schematic representation of interaction between ligands (compounds **8** and **18**) and BChE before the MD simulation. HOH806, HOH765, HOH749, HOH761, and HOH715 were conserved as structural water molecules. (A) **8**; (B) **18**.

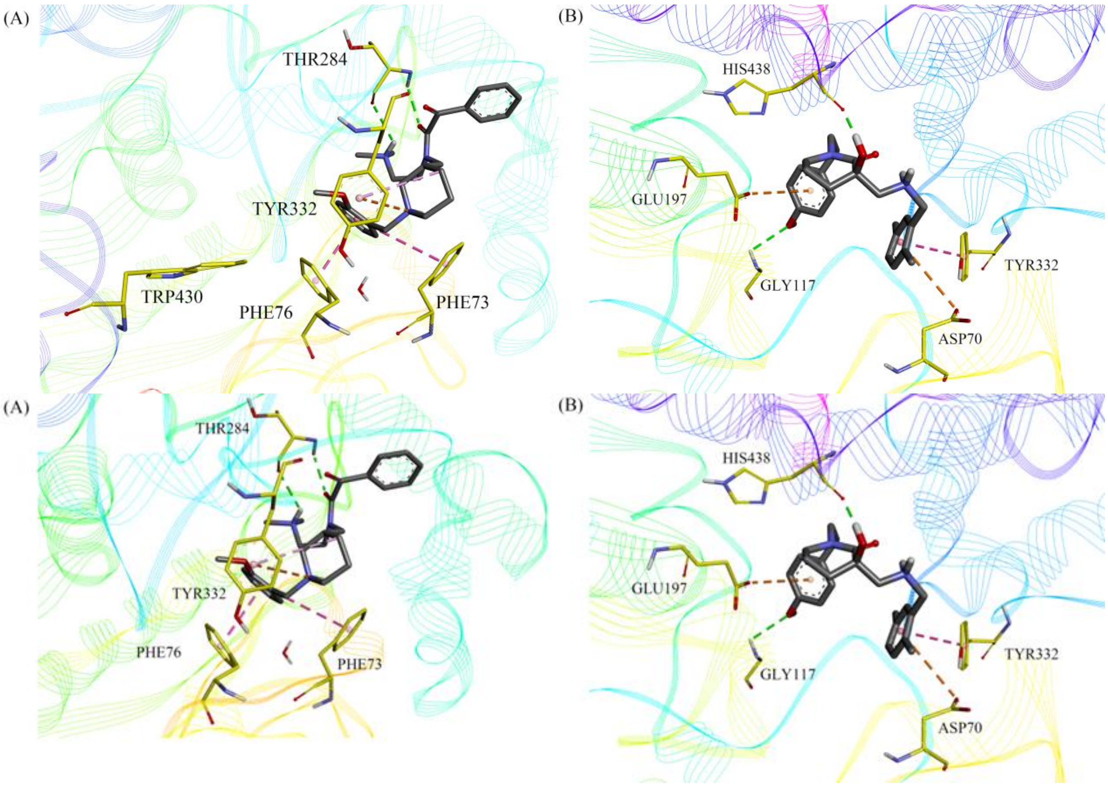

Figure S4. The binding modes of ligands (compounds **8** and **18**) with BChE (PDB ID: 5DYW). Protein models were taken from the last frame of the MD simulation. (A) **8**; (B) **18**. Compounds are shown in grey stick mode; key residues are shown in yellow line modes; π-π stacking is depicted in pink dot line; H-bond is in green dot; π-anion is in orange dot line.
